# Characteristics and outcomes of lung cancer patients requiring ventilatory support: results from a multinational study

**DOI:** 10.1186/cc14621

**Published:** 2015-03-16

**Authors:** AC Toffart, JF Timsit, J Salluh, G Burghi, C Irrazabal, N Pattison, E Tobar, B Almeida, E Azoulay, M Soares

**Affiliations:** 1 Hopital A. Michalon CHU Grenoble, France; 2 Post-graduation Program - Inst. Nacional de Cancer, Rio de Janeiro, Brazil; 3 Hospital Maciel, Montevideo, Uruguay; 4 Instituto Alexander Fleming, Buenos Aires, Argentina; 5 Royal Brompton Hospital, London, UK; 6 Hospital Clinico Universidad de Chile, Santiago, Chile; 7 Hospital A.C. Camargo, São Paulo, Brazil; 8 Hopital Saint Louis, Paris, France; 9 DOr Institute for Research and Education - IDOR, Rio de Janeiro, Brazil

## Introduction

The aim was to evaluate clinical characteristics and outcomes of patients with lung cancer requiring ventilatory support.

## Methods

Secondary analysis of a prospective multicenter study including patients requiring either invasive (IMV) or non-invasive (NIV) mechanical ventilation for >24 hours admitted to 22 ICUs in six countries from Europe and South America during 2011. We used shared frailty models to identify factors associated with 6-month survival.

## Results

Out of 449 patients admitted to the ICUs, 239 (small-cell (SCLC) = 34; non-SCLC = 205)) required ventilatory support (NIV = 104; IMV = 135). Out of NIV patients, 31 (30%) were subsequently intubated for IMV. Main reasons for ventilatory support were sepsis (*n *= 119; among them, 102 patients had pneumonia), airway involvement by tumor (*n *= 79), ARDS (*n *= 47) and coma (*n *= 18). Mean SAPS II score was 54 ± 20 and median SOFA score was 7 (4 to 12) points. Hospital and 6-month mortality rates were 55% and 67%; 94 (39%) patients received treatment limitations in the ICU. Mortality according to ventilatory strategy was 56% for NIV only, 77% for NIV followed by IMV, and 70% for IMV only. In the multilevel model, adjusting for the hospital size, presence of step-down units, type of admission and treatment limitation, performance status (PS) 3 to 4 (HR = 2.25 (95% CI, 1.52 to 3.34)), metastasis (HR = 1.66 (1.18 to 2.33)) and the ventilatory strategy compared with NIV only (HR = 1.73 (1.02 to 2.92), for NIV followed by IMV; HR = 2.25 (1.51 to 3.35), for IMV only) were associated with increased mortality. Conversely, patients with sepsis had higher survival (HR = 0.67 (0.46 to 0.96)).

## Conclusion

In a multinational study, 6-month survival in lung cancer patients requiring ventilatory support is better than perceived *a priori*. Palliative care should be preferred in patients with poor PS.

